# Optical characterization of deuterated silicon-rich nitride waveguides

**DOI:** 10.1038/s41598-022-16889-7

**Published:** 2022-07-26

**Authors:** Xavier X. Chia, George F. R. Chen, Yanmei Cao, Peng Xing, Hongwei Gao, Doris K. T. Ng, Dawn T. H. Tan

**Affiliations:** 1 Photonics Devices and Systems Group, Engineering Product Development, Singapore University of Technology and Design, 8 Somapah Road, Singapore, 487372 Singapore; 2 Institute of Microelectronics, Agency for Science, Technology and Research (A*STAR), 2 Fusionopolis Way, Singapore, 138634 Singapore

**Keywords:** Engineering, Optics and photonics

## Abstract

Chemical vapor deposition-based growth techniques allow flexible design of complementary metal-oxide semiconductor (CMOS) compatible materials. Here, we report the deuterated silicon-rich nitride films grown using plasma-enhanced chemical vapor deposition. The linear and nonlinear properties of the films are characterized, and we experimentally confirm that the silicon-rich nitride films grown with SiD_4_ eliminates Si–H and N–H related absorption. The performance of identical waveguides for films grown with SiH_4_ and SiD_4_ are compared demonstrating a 2 dB/cm improvement in line with that observed in literature. Waveguides fabricated on the SRN:D film are further shown to possess a nonlinear parameter of 95 W^−1^ m^−1^, with the film exhibiting a linear and nonlinear refractive index of 2.46 and 9.8 $\times$ 10^–18^ m^2^W^−1^ respectively.

## Introduction

Chemical vapor deposition (CVD) tools allow flexible growth of thin films. Through a judicious selection of precursor gases, flow rates and process temperatures, films with varied optical properties may be designed and realized. Amongst the spectrum of materials which may be grown using CVD techniques, silicon and silicon–nitride based materials are of particular interest for photonics and electronics. These materials are compatible with complementary metal oxide semiconductor processes and used in a plethora of applications. Amorphous silicon is a commonly used material for solar cells^1^ whereas silicon nitride is a commonly used passivation layer in electronics^2^. With regards to photonics, CVD grown silicon and silicon–nitride based materials possess favorable optical properties such as high transmissivity at the visible (silicon nitride), short-wave infrared (silicon and silicon nitride) to the mid-infrared (silicon and silicon nitride) wavelengths^3–5^, importantly also being compatible with complementary metal-oxide semiconductor (CMOS) processing. Silicon-rich nitride is a material whose properties may be engineered to possess the optical properties of both Si and Si_3_N_4_. Indeed, significant progress has been made in silicon-rich nitride based nonlinear optics in recent years, largely due to the high nonlinear refractive index and absence of two-photon absorption at the telecommunications wavelengths.

Silicon-rich nitride films require precursor gases containing silicon and nitride atoms for their growth. Commonly used precursor gases supplying the silicon content are silane (SiH_4_) and dicholorosilane (SiH_2_Cl_2_), whereas nitrogen (N_2_) gas and ammonia (NH_3_) supply the nitrogen content. From the standpoint of photonics applications, N_2_ is a superior precursor gas than NH_3_, as it precludes formation of N–H bonds which also possess absorption overtones at the short-wave infrared wavelength^6^. In our prior work, we have aimed to eliminate N–H bonds in ultra-silicon-rich-nitride films by utilizing N_2_ gas instead of NH_3_ gas^7–9^. The formation of Si–H bonds in films however is not easily resolved whether SiH_4_ or SiH_2_Cl_2_ precursor gases are used for the film growth. Consequently, Si–H absorption overtones at the shortwave infrared are a source of absorption in photonic devices^10^. To overcome Si–H absorption, the Si_3_N_4_ community routinely anneals their films at temperatures up to 1200 °C^11,12^. This allows ultra-low loss films to be created. An alternative method of reducing Si–H related loss involves using precursor gases which do not contain any Si–H bonds. Deuterated silane (SiD_4_) is chemically almost identical to SiH_4_, with the exception that the H atoms are replaced by deuterium, an isotope of hydrogen. Si–D bonds have been reported to have an absorption overtone close to 2 μm as opposed to close to 1.55 μm in the case of Si–H^13^. Consequently, growth of films using SiD_4_ allows an elegant and straightforward solution to eliminating Si–H related absorption. Deuterated silane has been used with significant success in Hydex glass, which has spawned a multitude of nonlinear optics advancements over the years^14–16^, as well as in the growth of SiO_2_, SiON, Si_3_N_4_ and amorphous silicon films^17,18^. In a similar vein, O–H bonds which form during optical fiber manufacturing have historically posed a problem due to their absorption being located at the O-band. The replacement of O–H bonds with O–D bonds has also been shown to allow significant loss reductions at the O-band^19^. In practice, the optical fiber is exposed to an atmosphere of deuterium during the manufacturing process to reduce the hydrogen related attenuation^20^. The use of deuterium is therefore advantageous in many respects for photonics applications.

In this manuscript, we report the growth and optical characterization of silicon-rich nitride films grown using SiD_4_ gas (SRN:D). Materials characterization using Fourier Transform Infrared Spectroscopy verify the absence of Si–H bonds in the films grown using plasma enhanced chemical vapor deposition. Waveguide devices are fabricated using the film and the linear and nonlinear properties are reported. Propagation losses are compared with a conventional SRN film fabricated with identical deposition parameters, demonstrating a 2 to 2.5 dB/cm improvement using SRN:D films. We achieve a nonlinear refractive index of 9.8 $\times$ 10^–18^ m^2^W^−1^, an order of magnitude larger than in stoichiometric silicon nitride, and a waveguide nonlinear parameter of 95 W^−1^ m^−1^. The films are further characterized to have a bandgap of 1.9 eV, indicating that two-photon absorption is absent at a wavelength of 1.55 µm.

## Film characterization

The fundamental vibrational absorption for Si–H bonds has been reported to occur at the wavenumber region at around 2200 cm^−1^^21–25^. The third vibrational overtone which resides at the region around 6600 cm^−1^ (1.5 μm wavelength region) is responsible for the deleterious attenuation associated with Si–H bonds at telecommunications wavelengths. The quantum harmonic oscillator model may be used to describe the vibrational bond energies associated with Si–H bonds^26^:

1 $$-\frac{{h}^{2}}{2m}\frac{{d}^{2}\psi }{{dx}^{2}}+\frac{1}{2}k{x}^{2}\psi =E\psi$$

where *h* is Planck’s constant, *Ψ* is the wave function, *x* is the displacement from equilibrium and *E* is energy. Enforcing the boundary conditions, *Ψ* = 0, $x=\pm \infty$, generates the closed form expression for the vibrational bond energy as given by^26^:

2 $${E}_{\upsilon }=h.\left(1/2\pi \right).\left(\upsilon +1/2\right)\sqrt{k/{m}_{1}+k/{m}_{2}}$$

where $\upsilon$ is the vibrational quantum number (an integer), *k* is the bond’s force constant and *m*_*1,2*_ is the atomic mass for the two atoms making up the bond. Referring to the fundamental vibrational absorption for Si–H bonds in the context of Eq. (2), it may be observed that a change in m_1_ or m_2_ will result in a change in the vibrational bond energy. Given that the atomic mass of deuterium (D), hydrogen (H) and silicon (Si) is 1, 2 and 28 respectively, the fundamental vibrational bond energy for Si–H is expected to decrease with the substitution of H with the D atom. From Eq. (2), it may be derived that Si–H’s third vibrational absorption overtone which resides at the region around 6600 cm^−1^ (1.5 μm region) will be reduced to 4670 cm^−1^ (2.1 μm region). Consequently, the replacement of Si–H bonds with Si–D bonds by using deuterated silane instead of silane gas generates a significant red-shift in the Si–H absorption associated with the vibrational bond energy at the telecommunications wavelength region, such that any material related absorption arising from Si–H will be eliminated.

The SRN: D films are grown using plasma enhanced chemical vapor deposition at a temperature of 350 °C. To fabricate the SRN:D films, deuterated silane (SiD_4_) and N_2_ gas were used in lieu of conventional silane (SiH_4_) and NH_3_. In the deposition, we utilized a SiD_4_ and N_2_ flow rates with a ratio of 3:250. Films with a thickness of 310 nm were then deposited on a silicon substrate with a 5 μm thermal SiO_2_ layer that would form the lower cladding of deposited devices later during device fabrication. The 5 µm film thickness of the thermal oxide layer ensures that any leakage of the propagating fields into the silicon substrate is prevented. The use of deuterated silane and N_2_ replaces the Si–H and N–H bonds with Si–D and N–D respectively, resulting in the shifting of bond absorbance overtones away from the telecommunications region at 1.55 μm^6,13,25^.

**Figure 1:**
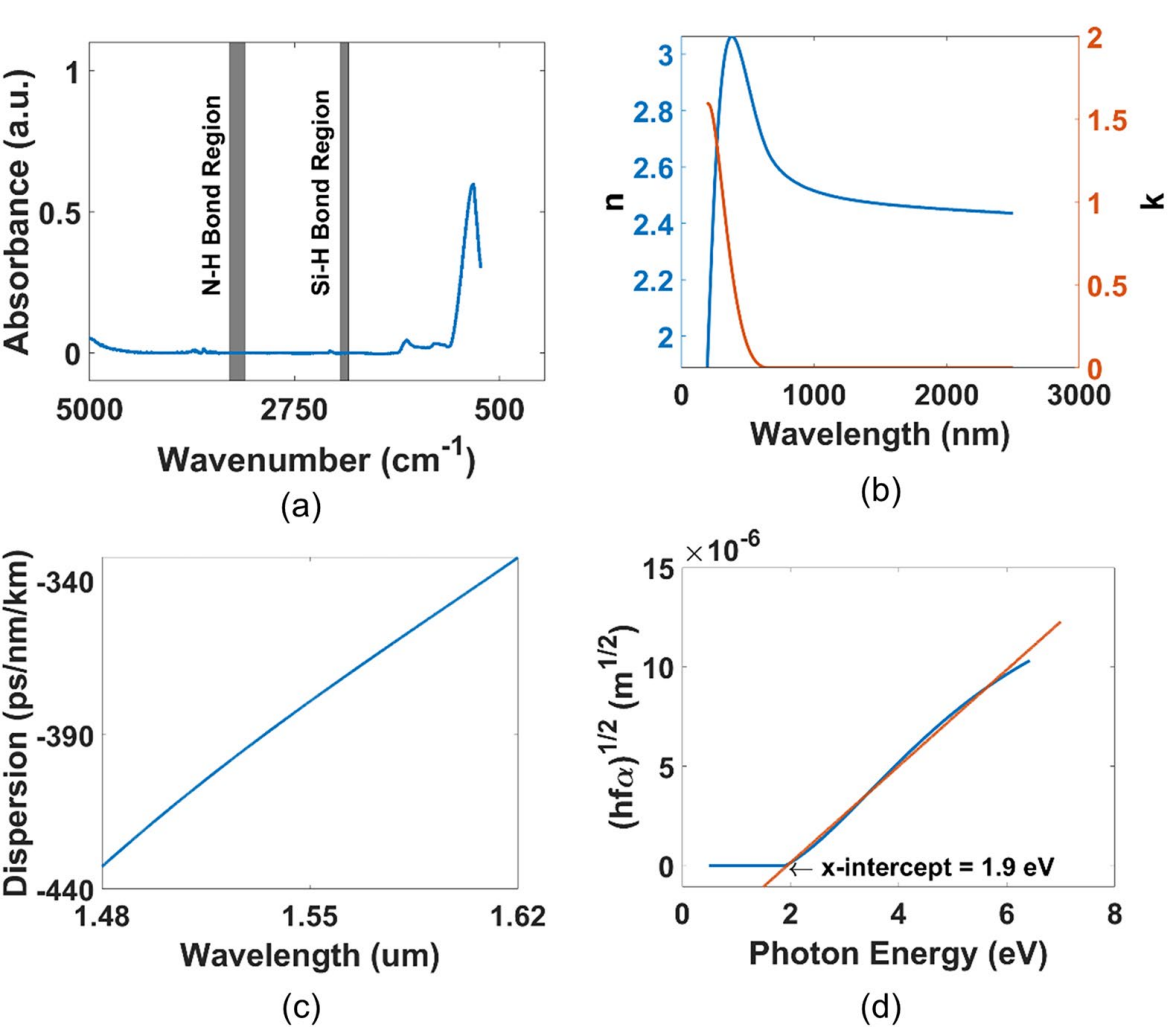
(**a**) Baseline-adjusted absorbance plot exhibiting regions where Si–H and N–H bonds could occur. We note no peak occurrences in these regions, indicating absence or negligible Si–H and N–H bonds. (**b**) Refractive index n (﻿) and extinction coefficient k (﻿) data extracted using FTIR measurements (**c**) Material dispersion curves from 1.48 to 1.62 µm for the SRN-D film (**d**) Tauc’s plot of the SRN-D film with the original curve (﻿) and fitted line (﻿).

We first characterize the material properties of the SRN:D film. Fourier-Transform Infrared (FTIR) spectroscopy was conducted to determine the material absorption of the deposited films (Fig. 1a), which sees the illumination of the film with a broadband infrared source, allowing for the quantification of bonds present in the film based on the absorption spectra. Regions where Si–H bonds and N–H bonds could occur^21–24^ are highlighted at wavenumber range 2157 cm^−1^ to 2250 cm^−1^ and 3290 cm^−1^ to 3464 cm^−1^ respectively. We note that there are no peak occurrences in these regions, indicating that Si–H and N–H absorption bonds are absent or negligible. In conventional silicon-rich nitride films using SiH_4_-based gas precursors, peaks are easily observed in the absorbance spectra corresponding to Si–H and N–H bonds^21,23^, evidencing the presence of Si–H and N–H absorption bonds. We further investigate the FTIR data, specific at Si–H bond and N–H bond regions to quantify the Si–H and N–H bonds. Small peaks are identified at wavenumbers ~ 2200 cm^−1^ and ~ 3350 cm^−1^ where Si–H and N–H bonds are likely to occur^24^. We quantify the bond concentrations by relating the areas of the Si–H and N–H absorbance lines with the concentrations of Si–H and N–H bonds using Lanford and Rand’s technique^27^, with equations^21,24,27^:

3 $$\left[Si-H\right]=\frac{1}{2.303\times {\sigma }_{Si-H}}\times {\int }_{band}\alpha \left(\omega \right)d\omega ,$$

4 $$\left[N-H\right]=\frac{1}{2.303\times {\sigma }_{N-H}}\times {\int }_{band}\alpha \left(\omega \right)d\omega .$$

Here, ${\sigma }_{Si-H}=7.4\times {10}^{-18} {cm}^{2}$ is the absorption cross-section of Si–H and ${\sigma }_{N-H}=5.3\times {10}^{-18} {cm}^{2}$ is the absorption cross-section of N–H, $\int \alpha \left(\omega \right)d\omega$ is the baseline absorption area of the band. In addition,

5 $$\alpha =\frac{2.303}{t}A$$

Here, $A$ is the absorbance and $t$ is the film thickness. Si–H bond concentration is calculated to be $1.17\times {10}^{20} {cm}^{-3}$ and N–H bond concentration is calculated to be $9.36\times {10}^{19} {cm}^{-3}$. Compared to Si–H and N–H bond concentrations reported in literature^21,24^ using SiH_4_-based gas precursors which calculate the bond concentrations to be in the range of ${10}^{21}$–${10}^{22} {cm}^{-3}$, the Si–H and N–H bond concentrations in our SRN:D film are 1–2 orders of magnitude smaller, further confirming the effectiveness of the SiD_4_ gas in significantly reducing Si–H bonds.

The *n* and *k* data were also extracted by fitting data generated from ellipsometry (Fig. 1b), revealing a linear refractive index of 2.46 at a wavelength of 1.55 µm. Extinction coefficient ($k$) values reveal good broadband transparency. Following this, the material dispersion for the film was calculated (Fig. 1c)^28^ and it is observed that the material dispersion is normal at the 1.55 µm wavelength region.

Subsequently, Tauc’s Method^29^ was applied to determine an optical bandgap of 1.9 eV. Tauc’s Method is derived from the governing expression relating absorption coefficient of an amorphous material, $\alpha$ with its frequency and bandgap^30^

6 $${\left(hf\alpha \right)}^\frac{1}{2}=B(hf-{E}_{g})$$

Here, $h$ is Planck’s constant, $f$ is the frequency of the incident photon, $r$ is the nature of the electron transition, $B$ is a material-dependent constant, and ${E}_{g}$ is the bandgap of the material. The optical bandgap of the material can be extracted by plotting ${\left(hf\alpha \right)}^\frac{1}{2}$ vs $hf$ and extrapolating a fit of the linear portion of the to determine the x-intercept. The absorbance $\alpha$ can be determined from the $k$ data in Fig. 1b using $\alpha =\frac{4\pi k}{\lambda }$. Figure 1d shows the Tauc plot from the 310 nm SRN:D film and the optical bandgap is determined to be 1.9 eV.

The optical bandgap of a medium determines the wavelengths at which non-negligible material losses are present in the film^31^. A bandgap of 1.9 eV corresponds to a wavelength of 640 nm, indicating that the SRN-D film is well suited for applications within the telecommunications bands at 1.55 µm. An optical bandgap of 1.9 eV also precludes the effects of two-photon absorption (TPA) around 1.55 µm which can be observed amorphous silicon devices, which makes SRN:D a suitable medium for nonlinear applications in the telecommunications band.

## Device characterization

**Figure 2:**
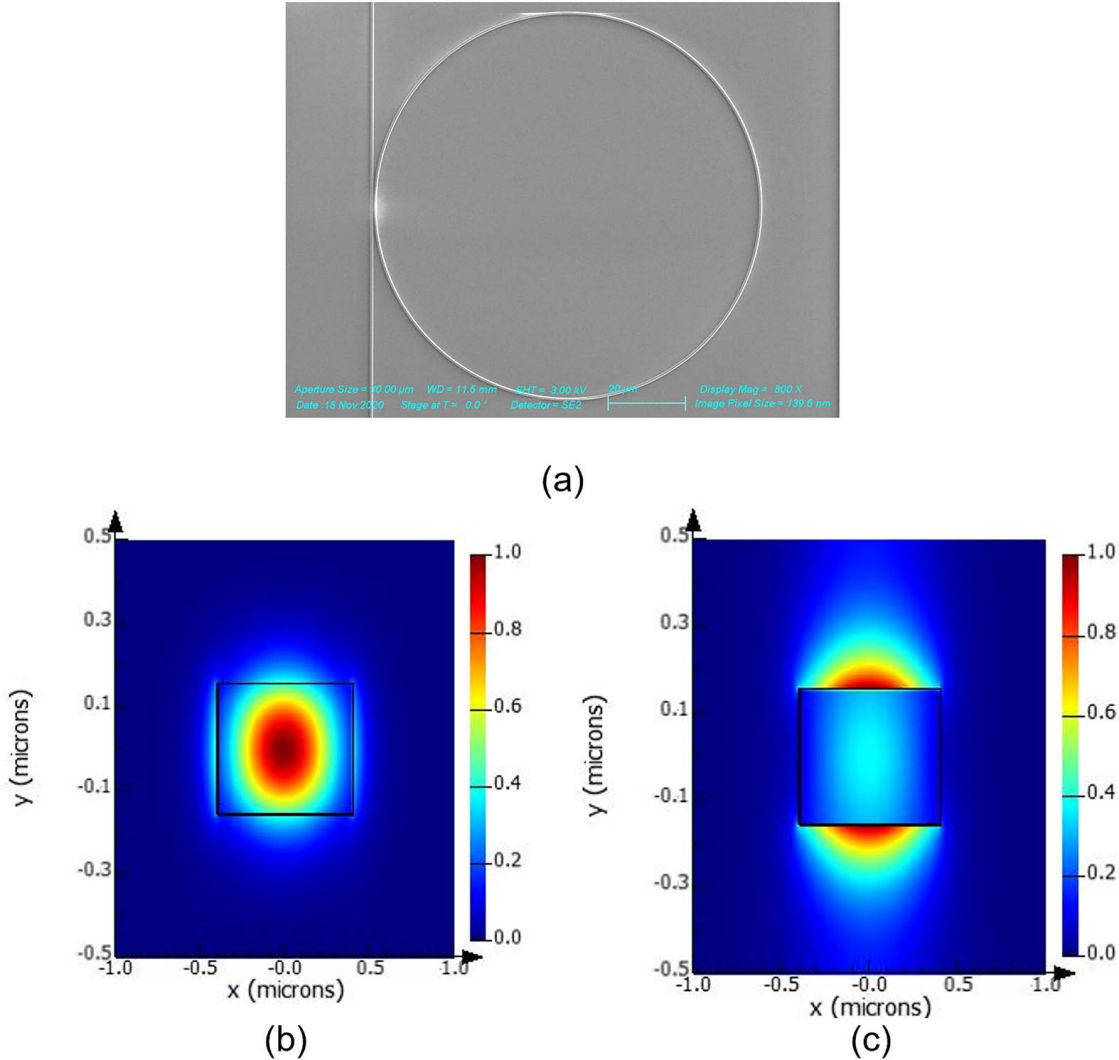
(**a**) SEM Micrograph of all-pass ring resonator (**b**) Mode Profile of Quasi-TE mode at 1.55 µm (**c**) Mode Profile of quasi-TM Mode at 1.55 µm.

The characterisation of linear optical properties of the film necessitates the fabrication of waveguides and resonators. A series of waveguides, microring, and racetrack resonators were patterned using electron-beam lithography and etched with reactive ion etching, following which a 2 μm thick layer of SiO_2_ was deposited to form the upper cladding. Figure 2a shows a scanning electron micrograph of a fabricated ring resonator. Figure 2b,c further show the mode profiles for the quasi-TE and quasi-TM modes of the waveguide.

**Figure 3:**
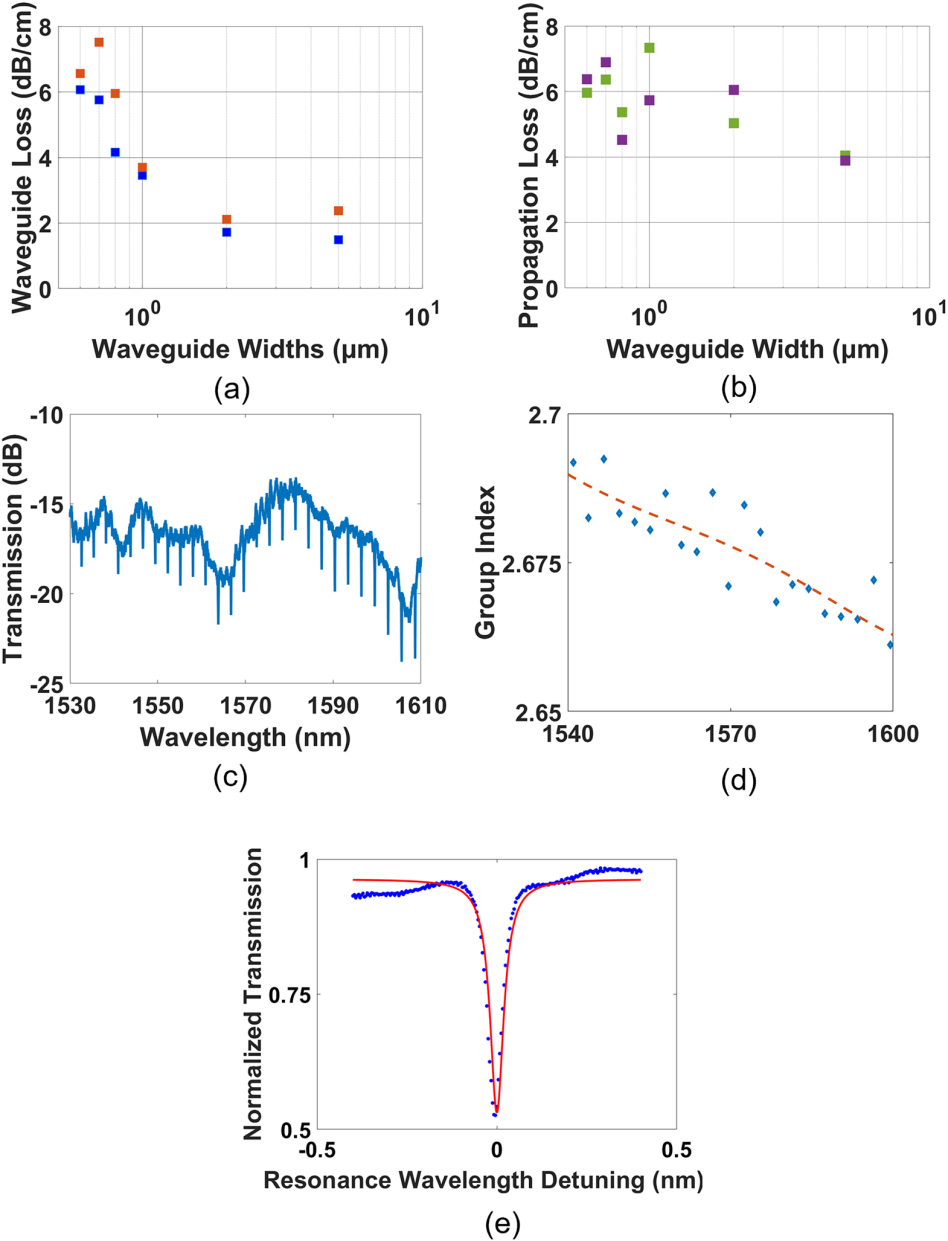
(**a**) Waveguide propagation losses from cutback measurements on the SRN:D platform for TE (blue filled square) and TM (orange filled square) Modes (**b**) Waveguide propagation losses from cutback measurements on the conventional SRN platform (fabricated using SiH_4_) for TE (green filled square) and TM (violet filled square) Modes (**c**) Transmission spectrum for microring resonator of radius 50 µm and gap 300 nm for the quasi-TE mode. (**d**) Experimental (blue filled diamond) and fitted () Group Index extracted from microring resonator of radius 50 µm and gap 300 nm (**e**) Lorentzian fit showing experimental data of a resonant dip (blue open circle) and fitted curve ().

A broadband tunable single-mode continuous wave laser source was edge-coupled using polarization-maintaining fibers into the devices. To determine the propagation loss for each waveguide width, cutback measurements were conducted and the results are shown in Fig. 3a. In both TE and TM modes, the propagation loss is observed to decrease with an increase in waveguide width across the same film thickness, indicating that sidewall roughness is a key contributor to propagation loss given the increased side wall interaction within the smaller waveguide widths studied^32^. This observation is further reinforced by the generally lower propagation losses in TM modes for waveguides of smaller width, as may be attributed to the decreased sidewall interaction in the fundamental TM mode (Fig. 2c) compared to the TE mode (Fig. 2b).

In the results from the cutback measurements shown in Fig. 3a, the propagation losses in the TE mode are observed to asymptotically approach values of 1.5 dB/cm with increasing waveguide widths, a value comparable to SRN films with similar film thickness and refractive index^33^. The decreasing propagation loss observed as waveguide widths increase is indicative of sidewall roughness being a dominant loss mechanism. For SRN, these loss values are considerably lower, with propagation loss limits closer to 2.5 dB/cm being commonly reported^13^. Given the replacement of Si–H and N–H bonds from the film shown in Fig. 2a, material absorption is a negligible contributor to the propagation losses and key contributors to the 1.5 dB/cm can thus be concluded to arise from surface roughness during the deposition process or sidewall roughness induced during the lithography and etch processes. With additional process optimization, contributions to the propagation losses from these mechanisms can be further reduced.

To compare the loss performance of the devices against those fabricated on a conventional SRN platform, a film is deposited using identical parameters using conventional silane (SiH_4_) and nitrogen gas (N_2_). The deposition of this film allows for a direct comparison between both platforms. Waveguides of identical widths are patterned and realised using the same recipe, and their propagation losses characterized using the same method. Results of the experiments are plotted in Fig. 3b. Compared to the SRN:D devices, the structures fabricated on conventional SRN approach a propagation loss of ~ 4 dB/cm (at wider waveguide widths) for both the TE and TM propagation modes, which places their performance at roughly 2–2.5 dB/cm higher compared to the presented SRN:D devices. The propagation losses are higher in the SiH_4_ sample, and this effect is particularly pronounced when the waveguide widths are larger. In the presence of sidewall roughness induced by the lithography and etching process, propagation losses will possess a component from material losses and from the sidewall roughness. The loss contribution from sidewall roughness will dominate at smaller waveguide widths where the mode is more confined, whereas material loss will dominate at wider waveguide widths where the mode is less confined. It is clear from Fig. 2b that the propagation loss in the regime where material losses dominate (in the limit of wide waveguide widths), is substantially higher for the SiH_4_ devices. For instance, the propagation loss for a waveguide width of 5 μm is 2 dB higher in the SiH_4_ devices than the SiD_4_ devices. This result is in good agreement with reported limits in literature^13,34^, and highlights the effectiveness of SRN:D in reducing losses for devices operating near 1.55 µm.

The source was similarly coupled into SRN:D resonators, following which a benchmark attainable intrinsic quality factor and the group index of these waveguides could be determined. Figure 3c shows the transmission spectrum of the microring resonator of radius 50 µm and gap 300 nm when quasi-TE light is coupled into the device. The free spectral range (FSR) of the resonators can be obtained as follows^35^:

7 $$\mathrm{FSR}=\frac{{\lambda }_{0}^{2}}{{n}_{g}L}$$

Here, ${\lambda }_{0}$ is the resonant wavelength, ${n}_{g}$ the group index, and $L$ the optical path-length of the resonator. From the transmission spectrum of the devices (Fig. 3c), the FSR at the resonances may be obtained by identifying the wavelength differences between each resonant dip and the group index of the devices experimentally obtained (Fig. 3d).

The loaded ($${Q}_{L}$$) and intrinsic ($${Q}_{int})$$ quality factors can also be obtained^36^:

8 $${Q}_{\mathrm{L}}=\frac{{\lambda }_{0}}{\mathrm{FWHM}},$$

9 $${Q}_{int}=\frac{2{Q}_{L}}{1+\sqrt{{T}_{0}}}.$$

Here, FWHM is the full-width at half-maximum of the resonant dips when fitted to a Lorentzian (Fig. 3e) and ${T}_{0}$ is the fractional transmission at the resonance. Of the fabricated devices, a microring resonator of waveguide width 0.85 μm and gap 0.3 μm yielded the highest loaded quality factor of 62,000, corresponding to an intrinsic quality factor of 64,700. In general, the quality factors of the ring resonators decrease with increasing wavelength, suggesting that the effective index of the devices are also lower at higher wavelengths, in line with observations made from the refractive index in Fig. 2b.

The waveguide dispersion of the devices can be determined by extracting the group index of the resonators from the FSR^28^:

10 $${\beta }_{2}=\frac{1}{c}\cdot \frac{d{n}_{g}}{d\omega },$$

11 $$D=-\frac{2\pi c}{{\lambda }^{2}}{\beta }_{2}=-\frac{2\pi }{{\lambda }^{2}}\left(\frac{d{n}_{g}}{d\omega }\right)=\frac{1}{c}\cdot \frac{d{n}_{g}}{d\lambda }.$$

Here, $c$ is the speed of light in a vacuum, $\omega$ is the angular frequency, $D$ is the dispersion in ps/nm/km, and ${\beta }_{2}$ is the 2nd order group velocity dispersion. The decreasing group index as a function of wavelength suggests that the dispersion of the fabricated waveguide shown in Fig. 3c is normal, which is similarly observed for lower film thicknesses in SRN devices. Anomalous dispersion is an important property for nonlinear optics phenomena such as efficient four-wave mixing and frequency comb generation, and while the fabricated films exhibit normal dispersion it is possible to engineer the waveguide geometry to bring about anomalous dispersion. However, the lack of anomalous dispersion in this film does preclude fabricated devices from realizing dispersion-dependent nonlinear effects such as frequency combs or supercontinuum generation, which are important for applications requiring wideband sources such as communication or spectroscopic applications.

**Figure 4:**
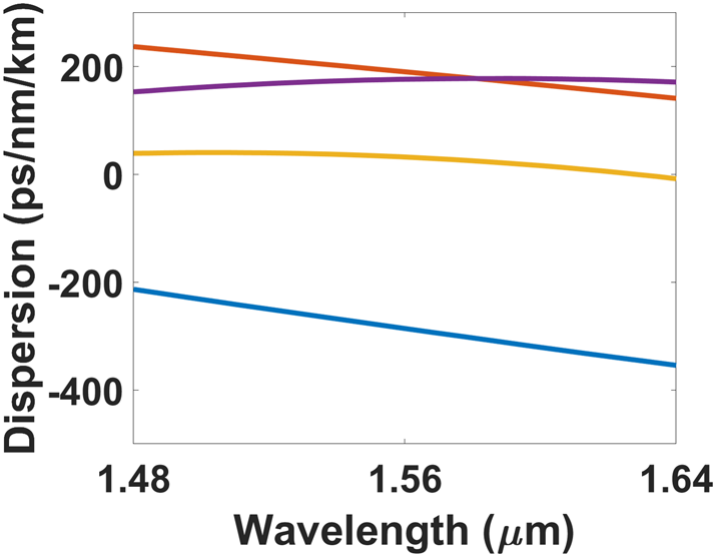
Simulated waveguide dispersion for waveguide width of 0.8 µm and film thickness 310 nm (), 400 nm (), 500 nm (), and 600 nm ().

Numerical simulations of various waveguide geometries were conducted to study the regime in which anomalous dispersion can be achieved. Figure 4 shows the results of these calculations and results suggest that the thickness of the SRN:D film needs to be increased beyond 500 nm to approach the required conditions for anomalous dispersion for the wavelength region of 1.48 to 1.64 µm.

The experimentally determined linear parameters such as the propagation loss, group indices, and dispersion of the devices also allow us to determine nonlinear parameters of the platform and the fabricated devices, providing insight into the suitability of the material as a potential platform for nonlinear optics. To characterize the nonlinearity of the waveguides, self-phase modulation (SPM) experiments were conducted. The maximum nonlinear phase-shift of pulses undergoing SPM (${\phi }_{NL})$ is dependent on the nonlinear parameter ($\gamma$) of the waveguides and the effective length (${L}_{eff}$). The governing expressions for these parameters are given by^28^:

12 $${\phi }_{NL}=\gamma {P}_{0}{L}_{eff},$$

13 $${L}_{eff}=\frac{\left[1-\mathrm{exp}\left(-\alpha L\right)\right]}{\alpha },$$

14 $$\gamma =\frac{2\pi {n}_{2}}{\lambda {A}_{eff}}.$$

**Figure 5:**
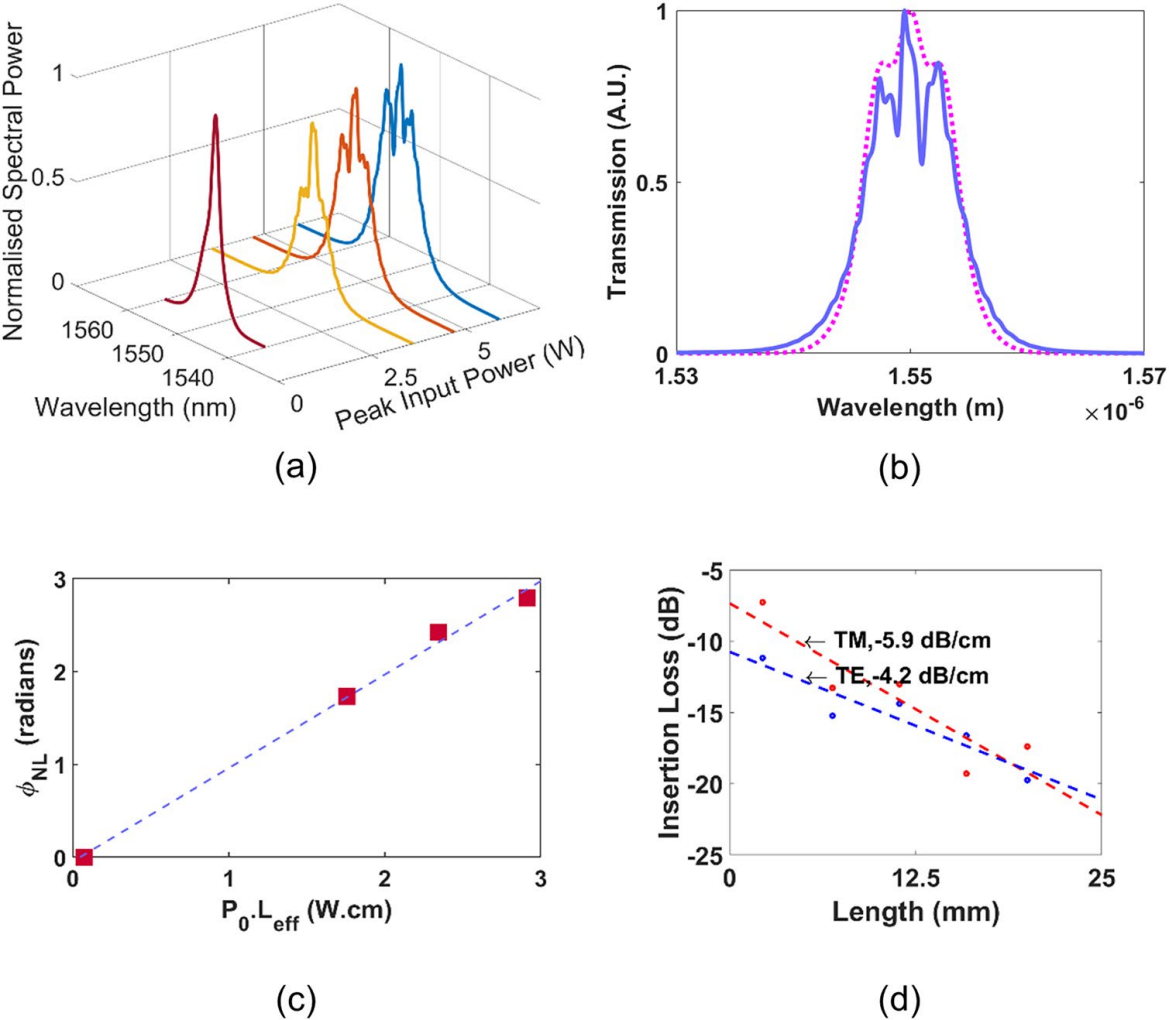
(**a**) SPM spectrum plots for peak input powers 3.6 W (), 4.7 W (﻿), 5.9 W (﻿), and Source (﻿) (**b**) Modelled (﻿) and experimentally measured () SPM spectra for ${P}_{0}$ = 5.9 W. (**c**) Parameter fit for experimental data (red filled square) and fitted line (). (**d**) Cutback measurements for the waveguide used for the SPM measurements, for both the TE (blue filled circle) and TM mode (red filled circle).

Here, ${P}_{0}$ is the peak input power, $L$ is the waveguide length in cm, $\alpha$ is the propagation loss in dB/cm, ${A}_{eff}$ is the effective modal area, and ${n}_{2}$ is the Kerr nonlinearity of the film. The SPM experiments were conducted by coupling 1.3 ps pulses from a fiber laser at a repetition rate of 20 MHz. Waveguides of width 0.8 µm and length 6.9 mm were chosen for their high modal confinement and lower propagation loss, and the input power was varied by displacing the input fiber. The variation of input power produces a series of spectra due to variations in the SPM process, which allows for a quantification of the waveguide nonlinear parameter. Figure 5a shows the evolution of the pulse spectrum as the input power is increased, reaching a maximum observed phase shift of 0.8 $\uppi$ at a peak pulse power of 5.9 W.

The nonlinear parameter $\gamma$ is extracted by plotting the SPM phase shift against the peak input power. Figure 5c plots the nonlinear phase shift as a function of *P*_0_*L*_eff_ where a linear relationship is obtained. From Eq. (12), the gradient of the plot in Fig. 5c allows us to extract a nonlinear parameter of $\gamma =95 {\mathrm{W}}^{-1}{\mathrm{m}}^{-1}$ for the waveguide. For a waveguide of 0.8 µm $\times$ 0.31 µm at a wavelength of 1.55 µm, the effective modal area ${A}_{eff}$ was determined to be 0.42 µm^2^, following which the Kerr nonlinearity was determined using Eq. (14) to be 9.8 $\times$ 10^–18^ m^2^W^−1^. The experimentally determined $\gamma$ and ${n}_{2}$ of the fabricated waveguides, are of values that are to that reported in crystalline silicon^37^. We model the SPM dynamics of the pulse propagation in the waveguide in order to corroborate the experimentally extracted nonlinear parameter. The pulse propagation dynamics may be described using the nonlinear Schrödinger equation^24^:

15 $$\frac{\partial A}{\partial z}=-\frac{\alpha }{2}A-i\frac{{\beta }_{2}}{2}\frac{{\partial }^{2}A}{\partial {t}^{2}}+i\gamma {\left|A\right|}^{2}A$$

where *z* is the longitudinal coordinate, *t* is time, *α* is the linear loss coefficient, *A* and *ω*_0_ are the slow varying pulse envelope and carrier frequency, respectively. Figure 5 (b) shows the modelled and experimental pulse spectrum at an input peak power of 5.9 W, where good agreement is achieved.

At a Kerr nonlinearity of 9.8 $\times$ 10^–18^ m^2^W^−1^, the SRN:D film possesses more than 5 times higher nonlinearity than SRN films reported in literature with similar film thickness and refractive index^33^ while retaining comparable propagation losses. This marks a notable improvement from presently available literature on SRN devices and stands to be further improved with further optimisation of the process steps to suit the material.

## Conclusion

We have fabricated silicon-rich nitride films using SiD_4_ and N_2_ precursor gases, allowing films to be grown without Si–H and N–H bonds, both of which are typically present in films fabricated using SiH_4_, SiH_2_Cl_2_ and/or NH_3_ gas. Consequently, bond overtones normally present at the telecommunications bands are absent, minimizing a key source of optical absorption and rendering the platform ideal for integrated optics applications around 1.55 µm. Using FTIR measurements, we confirm the absence of Si–H and N–H bonds within the films.

The optical bandgap of 1.9 eV extracted using Tauc’s method denotes the absence of two-photon absorption near 1.55 µm, making SRN:D a promising platform for power efficient nonlinear optical applications. Linear characterisation of the propagation losses also reveals an asymptotic approach towards losses as low as 1.5 dB/cm, which is comparable to best case values of PECVD deposited SRN devices in literature prepared without further annealing. A conventional SRN film was deposited using identical deposition parameters, and waveguides of matching parameters fabricated. Cutback measurements demonstrate a 4 dB/cm propagation loss in both the TE and TM modes, around 2.5 dB/cm higher than the SRN:D devices.

Calculations also suggest that anomalous dispersion could be achieved with larger film thicknesses, and further optimization of the film to reduce surface and sidewall roughness could yield lower propagation losses. This, coupled with the low inherent nonlinear loss of the films, could facilitate the realisation of frequency comb and supercontinuum generation at low peak pulse powers^13,38^. Further process optimization for achieving lower propagation losses may also be undertaken to optimize the smoothness of the etched surfaces.

Presently, the cost of fabricating the SRN:D devices is relatively more expensive as compared to conventional SRN, due largely to the increased expense of SiD_4_ compared to SiH_4_. However, the benefits of SRN:D are notable and not easily substituted with other materials. Subsequently, we believe that demand could drive supply and subsequently decrease the price of deuterated silane in the future.

In previous work, ultra-silicon-rich-nitride films demonstrated a nonlinear refractive index 100 times greater than stoichiometric silicon nitride^8,39–42^. Prevailing characterization of silicon–nitride thin films fabricated with different ratios of precursor gases suggests that the linear and nonlinear refractive indices of the SRN:D films increase correspondingly with silicon content^34,36,40,43^. Deuterated silicon-rich nitride films with optimized silicon content may therefore be a promising platform for a low-loss, CMOS-compatible, highly nonlinear integrated optics devices, importantly minimizing Si–H and N–H related absorption at the telecommunications bands.

## Data Availability

The datasets used and/or analysed during the current study are available from the corresponding author on reasonable request.
